# Exploring the Dark Matter of Human Proteome: The Emerging Role of Non-Canonical Open Reading Frame (ncORF) in Cancer Diagnosis, Biology, and Therapy

**DOI:** 10.3390/cancers16152660

**Published:** 2024-07-26

**Authors:** Anni Ge, Curtis Chan, Xiaolong Yang

**Affiliations:** Department of Pathology and Molecular Medicine, Queen’s University, Kingston, ON K7L 3N6, Canada; 21ag87@queensu.ca (A.G.); 18cbc4@queensu.ca (C.C.)

**Keywords:** human proteome, ncORF, micropeptide, microprotein, cancer biology, cancer diagnosis, cancer prognosis, cancer therapy

## Abstract

**Simple Summary:**

With advanced bioinformatic and sequencing technologies, many non-coding RNAs have been found to harbor non-canonical open reading frames (ncORFs). Some studies suggest that many ncORF-encoded microproteins or micropeptides possess important roles in cancer biology, yet the functional roles of these ncORF-encoded peptides are mostly unknown. The purpose of this review is to provide a comprehensive overview of existing knowledge on ncORF-encoded microproteins in cancer biology and highlight their roles as potential prognostic and therapeutic targets in cancer.

**Abstract:**

Cancer develops from abnormal cell growth in the body, causing significant mortalities every year. To date, potent therapeutic approaches have been developed to eradicate tumor cells, but intolerable toxicity and drug resistance can occur in treated patients, limiting the efficiency of existing treatment strategies. Therefore, searching for novel genes critical for cancer progression and therapeutic response is urgently needed for successful cancer therapy. Recent advances in bioinformatics and proteomic techniques have allowed the identification of a novel category of peptides encoded by non-canonical open reading frames (ncORFs) from historically non-coding genomic regions. Surprisingly, many ncORFs express functional microproteins that play a vital role in human cancers. In this review, we provide a comprehensive description of different ncORF types with coding capacity and technological methods in discovering ncORFs among human genomes. We also summarize the carcinogenic role of ncORFs such as pTINCR and HOXB-AS3 in regulating hallmarks of cancer, as well as the roles of ncORFs such as HOXB-AS3 and CIP2A-BP in cancer diagnosis and prognosis. We also discuss how ncORFs such as AKT-174aa and DDUP are involved in anti-cancer drug response and the underestimated potential of ncORFs as therapeutic targets.

## 1. Introduction

### 1.1. Definition of ncORF

Non-canonical open reading frames (ncORFs) are regions of translation that occur separately from the canonical protein-coding sequences [1]. In 2004, the Human Genome Project annotated the entire human genome and identified the location and sequences of around 20,000 protein-coding genes [2]. Historically, stringent rules were placed on the prediction of protein-coding open reading frames (ORFs) to minimize false positives [3]. For example, coding sequences (CDS) must meet a size cut-off of 100 amino acids; initiate translation from the conventional AUG start codon; and have monocistronic transcription, meaning each mRNA molecule cannot encode more than one polypeptide [3]. However, these rules created a blind spot in the human proteome for ncORFs that did not fit these criteria. Over the past two decades, improved understanding of gene expression and modern detection methods has rendered these criteria obsolete. To date, numerous ncORFs have been identified and are believed to have the potential to regulate gene expression or produce proteins with unique biological roles [4].

### 1.2. Classification of ncORF

**Figure 1:**
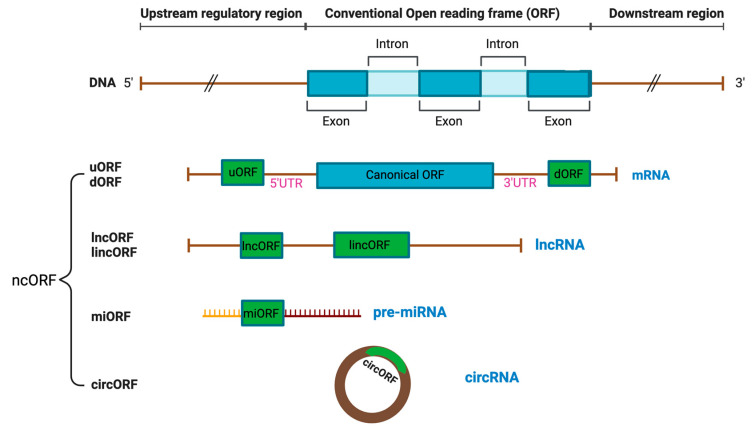
Different types of RNAs with coding capacity. Distinct categories of non-canonical open reading frames (ncORFs) have been identified from non-coding RNAs, which include 5′UTR upstream ORF (uORF), 3′ UTR downstream ORF (dORF), long non-coding ORF (lncORF), and long intergenic non-coding ORF (lincORF) from long non-coding RNAs (lncRNAs), miRNA ORF (miORF) from miRNA, and ORF derived from circular RNAs (circORF).

Technological advances have enabled the discovery of many neglected ncORFs, which are classified into different categories, such as long non-coding ORFs (lncORFs), circular ORFs (circORFs), intronic ORFs, primary microRNA-derived ORFs (pri-miORFs), and small ORFs (smORFs) (Figure 1) [1,3]. This section reviews ncORFs based on each category. 

ncORFs can be found within the 5′ and 3′ untranslated regions (UTR) of the annotated CDS in an alternate reading frame from the CDS, including upstream (uORFs) and downstream ORFs (dORFs), respectively (Figure 1) [3]. uORFs regulate translation of the main CDS through interactions with the 43S ribosome pre-initiation complex in the “leaky scanning” mechanism. As the 43S ribosome pre-initiation complex scans the mRNA and encounters the uORF, which often has a near-cognate non-AUG start codon, it has three options: (1) initiate translation at the uORF and disassemble upon reaching the stop codon, resulting in no translation of the downstream CDS; (2) fail to recognize the uORF and continue scanning until it reaches the downstream CDS, resulting in CDS translation; or (3) initiate transition at the uORF, remain bound to the mRNA, and resume scanning, resulting in CDC translation [3,5]. uORFs can encode proteins that interact or functionally cooperate with the downstream CDS protein [6]. A ncORF positioned at the upstream of a protein kinase C (PKC) isoform encodes uORF2, which significantly impairs viability of breast cancer and leukemia cells through inhibition of the PKC family [7]. Compared to uORFs, the function of dORFs is less defined, but it has been hypothesized that translation of dORFs can enhance main CDS translation by recruiting ribosomes or translation initiation factors to the main CDS [8]. Unlike uORFs and dORFs, alternative ORFs (altORFs) overlap with the main CDS, but in a shifted or alternate ORF [3]. The altFUS protein is a prime example of an altORF, where it overlaps its main CDS, FUS, in an open reading frame that is shifted by a single nucleotide [9]. ncORFs can also be in-frame with the main CDS, expressing extended or truncated isoforms of the annotated proteins based on the location and presence of the ncORF start and stop codons [3,10]. For example, the MYC gene has an alternate CUG start codon upstream of the main AUG start codon, which can translate into N-terminally extended variants of the MYC protein [11].

lncORFs are encoded within long non-coding RNA (lncRNA), which are RNA transcripts longer than 200 nucleotides annotated as non-coding (Figure 1) [3,12]. lncRNAs are pervasively expressed and play important roles in gene regulation as untranslated RNA molecules [13]. Many RNA sequences that are previously considered as lncRNA have now been discovered to contain ORFs with protein-coding potential [14,15,16,17,18,19,20]. An example of a lncORF is the steroid receptor RNA activator (SRA) gene. In its RNA form, SRA functions as a nuclear receptor coactivator [21,22], while it also encodes a protein, SRAP, associated with breast cancer cell motility [21,22]. lncRNAs can originate from pseudogenes, referring to DNA regions that contain presumably non-functional, untranslatable copies annotated genes derived from the retrotransposition of processed mRNAs and segmental duplication [12,23]. Increasing evidence has revealed peptides translated from pseudogenic ncORFs [24]. In the case of lncRNAs located in between genes with no overlap with the CDS, they are recognized as intergenic, and ncORFs derived from these regions are called long intergenic non-coding ORFs (lincORFs) or intergenic ORFs [25]. Though rare, intronic ORFs with translational capabilities have also been discovered in the introns of pre-sliced mRNAs [26]. Another type of non-coding RNA (ncRNA) is micro-RNAs (miRNAs) with 18–24 nucleotides, which originate as primary transcripts of miRNAs (pri-miRNAs) and become mature miRNAs through specific cleavage and progressing [27,28]. ncORFs derived from these unprocessed transcripts of miRNAs are referred to as pri-miORFs (Figure 1). 

Circular RNAs (circRNAs) are single-stranded RNA molecules covalently linked at the 5′ and 3′ ends as a product of back-splicing and display translation potential, resulting in the discovery of a new class of ncORFs, termed circORFs [3,29]. circORFs can regulate gene expression through their interactions with micro-RNA and circRNA binding proteins (cRBPs) [30]. Due to the lack of the 5′ end of linear messenger transcripts (5′ cap), circRNAs were historically considered to be a type of lncRNA [12]. However, through cap-independent initiation mechanisms, circRNAs have been demonstrated to encode functionally significant circORF proteins (Figure 1) [31]. An example of a circORF is circMAPK1, encoding MAPK1–109aa with tumor suppressive functions in gastric cancer cells through its interaction with MEK1 of the MAPK signaling pathway [32].

Finally, smORFs are sequences that encode micropeptides or microproteins with 100 amino acids or smaller in size [3]. This arbitrary size limit is due to the historical assumption that proteins smaller than 100 amino acids are statistically unlikely to be functional and the difficulty of detecting DNA sequences less than 300 nucleotides long before the emergence of modern omics-based technology, such as ribosome sequencing [3]. Regardless, all other classifications of ncORFs are also classified as smORFs if they meet the size cut-off. Outside of the nuclear genome, eight instances of smORFs have been identified in mitochondrial DNA, which encodes mitochondrial-derived peptides (MDPs) [33].

## 1.3. Identification of ncORF

### 1.3.1. Computational Techniques

Computational analyses have been the most common method for annotating canonical genes, where they are defined as the longest evolutionarily conserved AUG-containing ORF in an mRNA [34]. However, ncORFs/smORFs are trickier to predict and produce more noise, and thus alternative methods are required for their annotation [35]. The detection of ncORFs in the early stages was challenging due to the conservativeness of existing gene identification algorithms, which were not designed for ncORFs [36]. sORF finder is one of the first successful ncORF prediction tools, identificating over 2,000 intergenic ORFs in a plant species called Arabidopsis thaliana [37]. Technological improvements led to the establishment of various ncORF/smORF prediction tools and databases, such as PhyloCSF, MiPepid, uPEPperoni, and PhyloCSF [36,38,39,40,41]. In addition, machine learning approaches can be used to predict ncORFs based on features derived from genomic and transcriptomic data. Techniques such as support vector machines (SVMs) and neural networks can classify regions of the genome as coding or non-coding based on training data [42]. Databases such as Ensembl and UCSC Genome Browser provide resources for exploring ncORFs. These databases integrate data from multiple sources, including RNA-Seq and Ribo-Seq, and offer tools for visualizing and analyzing ncORFs in the context of the entire genome [43]. While bioinformatic tools provide great value in reduced cost and convenience compared to experimental validations of ncORFs, they are inadequate in confirming the translation of novel ncORFs [36]. Investigation and characterization of these translatable ncORFs require further study. 

### 1.3.2. Experimental Techniques

(a) Ribosome profiling: During translation, ribosomes enable protein synthesis by reading mRNA transcripts. As the ribosome reads the mRNA, it protects 30–31 nucleotides of the mRNA from nuclease degradation, creating a ribosome footprint. In 2009, Ingolia et al. exploited this phenomenon to develop the Ribo-Seq technique [44]. By converting these footprints to DNA sequences and utilizing deep sequencing, they were able to map the precise positions of ribosomes and quantify translational activity. Actively translated ORFs are characterized by continuous 3-nucleotide periodicity, which results from the 80S ribosomes reading the mRNA template one codon at a time [34]. Furthermore, ribosome footprint density can be used to deduce the rate of translation for a particular polypeptide, with higher ribosome density meaning slower elongation, and vice versa [45]. Another advantage of Ribo-Seq is its ability to identify ncORFs regardless of their start codon, which is invaluable since a large portion of ncORFs do not initiate translation with an AUG start codon [46,47]. Ribo-Seq data can be analyzed using computational methods, such as RibORF, which calculates the overall probability of translation [24,35]. However, validation of ncORFs at the protein level is required, as ribosome occupancy alone cannot distinguish between coding and non-coding RNA transcripts, limiting the reliability of Ribo-seq [36,48,49]. To reduce the number of false positives, a method called polysome profiling or Poly-Ribo-Seq was developed [50]. Scanning of the 40S ribosome subunit alone can result in ribosome footprints and the false detection of translation. Poly-Ribo-Seq takes advantage of the fact that polysomes, which consist of multiple ribosomes, bind to mRNAs collectively during active translation. By isolating polysomal fractions, active translation regions can be differentiated from false positives derived from single ribosomes or ribosome subunits. Yet, this is not a perfect solution as it leaves a blind spot for particularly short smORFs, which are not long enough to bind multiple ribosomes [50]. 

(b) Mass spectrometry (MS): MS-based proteomic techniques are often used to complement Ribo-Seq in the direct identification and quantification of ncORF-encoded proteins [36,51]. MS-based approaches can also aid in determining post-translational modifications, furthering our understanding of the protein’s functional mechanisms [52]. 

(c) CRISPR/Cas9 genome editing: the biological functions of ncORF-encoded proteins can be validated using CRISPR/Cas9-based approaches [36,53,54]. Knockout (loss-of-function) or overexpression (gain-of-function) assays can be performed to elucidate the functions of specific ncORF-encoded proteins [36,53,54]. The CRISPR/Cas9 system can also be used to observe the expression and localization of ncORF-encoded proteins by knocking-in epitope tags into DNA sequences of ncORFs, which can be detected by the corresponding antibodies [55]. Genome-wide CRISPR-based mutagenesis screens have successfully identified high-priority ncORFs that may encode functionally significant proteins such as GREP1, ASNSD1-uORF, or ASDURF [4,53,54]. CRISPR screening can also be combined with RNA sequencing (RNA-Seq) to confirm the biological function of ncORFs. By perturbing expression of candidate ncORFs using CRISPR/Cas9 and observing the changes in RNA-seq profiles within a single cell, the molecular mechanisms of the ncORF-encoded peptides can be readily elucidated [53]. 

## 2. ncORFs as Regulators of Cancer HallmarksIntroduction

**Figure 2:**
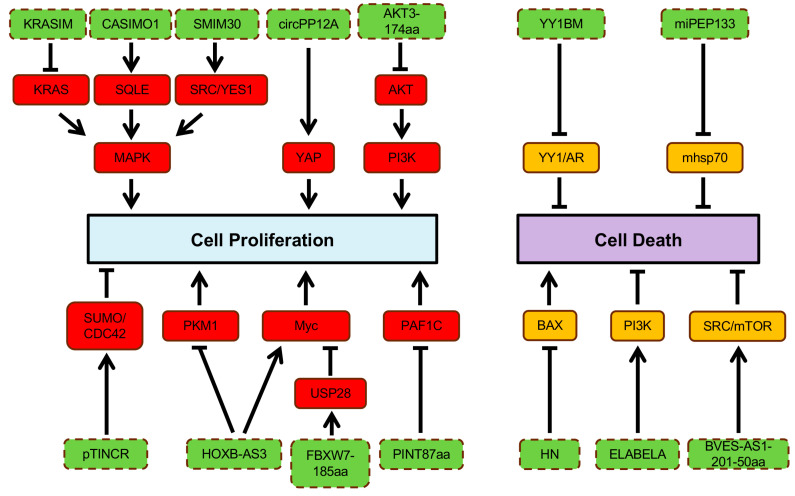
Regulation of cell proliferation and cell death by ncORF. ncORFs are indicated in blue color background. 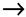 activation; 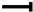 inhibition.

**Figure 3:**
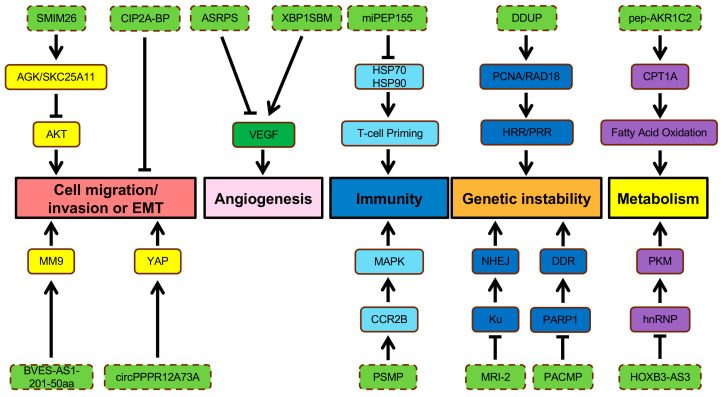
Regulation of cell migration/invasion, EMT, angiogenesis, immunity, genetic instability, and metabolism by ncORFs. Labels are as described in the legend of Figure 2.

Disruption of regulatory circuits that govern cell homeostasis enables the transformation of normal cells into malignant cancers, driven by genetic alternations [56]. Over two decades ago, Hanahan and Weinberg proposed six essential hallmarks acquired by the cancer cell genotypes, which were further extended to ten hallmarks including increased proliferation, reduced cell death, loss of tumor suppressor genes, increased angiogenesis, enabling replicative immortality, activating invasion and metastasis, immune escape, inflammation, genomic instability, and dysregulated metabolism [56,57]. Previous studies have shown that ncRNAs play important roles in regulation of various hallmarks of cancer [58]. In this review, we discuss the implication and molecular mechanism underlying the role of known ncORFs in different cancer hallmarks (Figure 2 and Figure 3). 

### 2.1. Cell Proliferation and Death

The development of cancer results in continual unregulated cell proliferation and inhibition of cell death [56]. Unlike normal cells, cancer cells can grow uncontrollably, invade normal tissues, and spread throughout the body, leading to metastasis [56]. This loss of growth control is a net outcome of abnormal cell regulatory pathways [56].

#### 2.1.1. Cell Proliferation

Normal cells are strictly regulated to inhibit uncontrolled proliferation [56]. However, cancer cells can grow uncontrollably by sustaining self-sufficient growth signals and evading growth suppressors [56]. Many proteins translated from ncORFs foster cell proliferation. For instance, a novel protein, Cancer-Associated Small Integral Membrane Open reading frame 1 (CASIMO1), is preferentially expressed in hormone-receptor-positive breast tumors and regulates proliferation and cell cycle [59]. CASIMO1 interacts with squalene epoxidase (SQLE) to induce ERK phosphorylation [59]. Loss of SQLE mimics the effect of CASIMO1 deficiency on breast cancer cell proliferation, indicating the role of CASIMO1 in modulating the MAPK pathway through its interaction with SQLE (Figure 2) [59]. These results suggest the oncogenic role of CASIMO1 in breast cancer cells. 

Additionally, the proliferation of HCC cells is modulated by a lncRNA-encoded peptide, SMIM30, which functions as an adaptor protein for membrane anchoring of non-receptor tyrosine kinases, SRC/YES1, to promote downstream MAPK signaling activation [60]. Another example is HOXB-AS3, a lncRNA-encoded peptide that exhibits opposing effects in different cancer types. Overexpression of the HOXB-AS3 protein enhances the proliferation and viability of oral squamous cell carcinoma cells through c-Myc mRNA stabilization [61]. Conversely, tumor growth of CRC is suppressed by the HOXB-AS3 peptide, which blocks glucose metabolic reprogramming through inhibition of the splicing of PKM1 (Figure 2) [62]. A ncORF found in C20orf204 RNA encodes C20orf204-189AA, which enhances proliferation of HCC cells when overexpressed; the mechanistic details of this peptide in promoting HCC-specific proliferation remain to be investigated [63]. The proliferative capability of cancer cells can also be promoted by other ncORF-encoded peptides, including ACLY-BP and circPPP1R12A-73aa [64,65]. Knockout of ACLY-BP significantly suppresses the proliferation of clear cell renal cell lines, suggesting its important role in maintaining the stability of ATP citrate lyase (ACLY), leading lipid metabolic shift to promote cell proliferation in clear cell RCC (ccRCC) (Figure 2) [65]. Increased proliferation ability was observed in colon cancer (CC) cells with circPPP1R12A-73aa overexpression, which subsequently activated YAP activity [64]. In addition, CRISPR knockout of 57 of the 553 ncORFs (10%) demonstrated a growth-inhibitory effect [54].

Although many proteins translated from ncORFs often display oncogenic roles in cancers, some peptides can exhibit tumor-suppressive roles. FBXW7-185aa, a novel protein encoded by the circular form of the FBXW7 gene, de-stabilizes cMyc by interacting with a de-ubiquitinating enzyme, USP28, to inhibit the proliferation and cell cycle acceleration in glioma cells (Figure 2) [66]. Two peptides encoded by circRNAs, AKT3-174aa and PINT87aa, have been discovered to suppress cell proliferation of GBM through two independent studies [67,68]. Specifically, AKT3-174aa negatively regulates the RTK/PI3K pathway by blocking AKT phosphorylation at the Thr308 site, and PINT87aa suppresses oncogenic transcriptional elongation through PAF1C [67,68]. The protein-coding capacity of *TINCR*, a ncRNA involved in epidermal cell differentiation, has recently been identified [69]. Expression of the pTINCR protein significantly suppresses the tumor growth through interaction with the SUMO/CDC42 complex in squamous cell carcinoma (Figure 2) [69]. KRASIM, another microprotein translated from lncRNA NCBP2-AS2, appears to reduce KRAS levels, suppressing ERK/MAPK signaling to inhibit the growth and proliferation of HCC cells (Figure 2) [70]. 

#### 2.1.2. Cell Death

The apoptotic program can be initiated by various physiological signals, triggering a series of choreographed molecular steps involving in disruption of cellular membranes, cytoskeletal collapsing, chromatin condensation, and nuclear fragmentation [56]. The main apoptotic pathways are extrinsic and intrinsic as well as an additional T cell mediated perforin/granzyme pathway [71]. Although each pathway depends on specific activating signals, they converge on the same execution pathway triggered by caspase-3 activation [71]. Tumor cells can gain apoptotic resistance by expressing anti-apoptotic proteins (e.g., Bcl-2) or inhibiting proapoptotic proteins (e.g., Bax) [71]. Many peptides translated from ncORFs have been implicated in cell death regulation. For example, humanin (HN) inhibits apoptosis by preventing Bax translocation from cytosol to mitochondria (Figure 2) [72]. Inhibition of HN expression resensitizes the cells to Bax [72]. One of lncRNAs, BVES-AS1, encodes a short peptide, named BVES-AS1-201-50aa, which promotes cell viability by activating Src/mTOR signaling in CRC cells [73]. ELABELA is a 54aa peptide hormone that activates PI3K/AKT signaling via the apelin receptor to stimulate hESC growth and maintain self-renewal through inhibition of apoptotic pathways (Figure 2) [19]. Another lncRNA-encoded microprotein regulating cell viability is YY1-blocking microprotein (YY1BM) [74]. Under nutrient deprivation, YY1BM induces apoptosis by inhibiting the interaction of YY1 and androgen receptor in male esophageal squamous cell carcinoma (Figure 2) [74]. As previously described, pri-miRNAs, precusors of miRNAs, have been found to harbor ncORFs that encode functional peptides [28]. Microproteins encoded by these ncORFs are termed miRNA-encoded peptides (miPEPs). A novel miPEP, miPEP133, has demonstrated its tumor suppressive ability [75]. Overexpression of miPEP133 induces cell cycle arrest and apoptosis by blocking the interaction between mitochondaril heat shock protein 70 and its binding partner (Figure 2) [75]. 

### 2.2. Metastasis

#### 2.2.1. Cell Migration and Invasion

Metastases account for 90% of cancer-related mortality [76]. The ability of a tumor to colonize foreign environments enhances its survival, especially when the nutrients and space become limited at the primary site [56]. Cell migration and invasion are initial steps during metastatic dissemination involving the activation of cellular pathways that modulate the dynamics of cytoskeletons in tumor cells [77]. The epithelial-to-mesenchymal transition (EMT) is an important cellular program activated by cancer cells to acquire invasive phenotypes during a metastatic event [78]. Loss of adherent junctions, acquisition of fibroblastic morphology, and expression of matrix-degrading proteases are common traits established during EMT [78]. A reversal of EMT traits occurs to recapitulate epithelial characteristics for colonization at a distant secondary place [78]. This process is often termed as mesenchymal to epithelial transition (MET) [78]. Regulation of F-actin dynamic is essential for cell motility, and loss of CASMO1 impacts actin assembly, dramatically reducing the migration ability of BC cells [59]. Moreover, two lncRNA-encoded peptides, SMIM30 and ZFAS1, independently promote cell migration and invasion of HCC cells [60,79], though their involvement of HCC metastatic events in vivo needs further exploration. BVES-AS1-201-50aa is one of many lncRNA-encoded proteins involved in tumor progression [73]. This 50 aa microprotein enhances the migratory and invasive abilities of CRC cells in vitro [73]. Overexpression of BVES-AS1-201-50aa is thought to increase the production of metastasis-associated protein MM9 in CRC cells (Figure 3) [73]. Through ribosome profiling and RNA-seq, a conserved and secreted peptide, Toddler, also known as APELA, is found to be expressed by a noncoding RNA in zebrafish, modulating cell movements during gastrulation [80,81]. Later research reveals that APELA is upregulated in ovarian cancer, contributing to cell migration and cancer progression [81]. Other than lncRNA-encoded proteins, circRNA is another type of ncRNAs containing ncORFs. One small peptide, circPPP1R12A-73aa, is expressed by such ncORFs from circPPP1R12A and displays metastasis-promoting abilities in CC by activating YAP activity (Figure 3) [64]. 

A lncRNA-encoded microprotein, SMIM26, behaves as a tumor suppressor by inhibiting EMT in ccRCC [82]. Overexpression of SMIM26 leads to reduced metastatic phenotypes, while silencing SMIM26 greatly enhances the migration and invasion of RCC cells [82]. Meng et al. (2023) revealed that SMIM26 interacts with AGK, a mitochondrial acylglycerol kinase, as well as glutathione transport regulator SKC25A11 to interfere with AKT-signaling-mediated metastasis (Figure 3) [82]. The role of TGF-β signal transduction is well recognized in cancer metastasis, and induced EMT phenotypes and cancer stemness can be achieved by TGF-β treatment in breast cancer [18]. CIP2A-BP is a microprotein encoded by lncRNA and inhibits metastatic capabilities when overexpressed in TNBC cells [18]. Expression of CIP2A-BP is usually reduced in TNBC by TGF-β signaling to induce tumor invasion and metastasis (Figure 3) [18]. Cell migration and invasion can also be inhibited by a previously discussed microprotein, miPEP133, though its anti-metastatic role in vivo has not been fully illustrated [75].

#### 2.2.2. Angiogenesis

Without blood supplies, nutrients and oxygen become depleted quickly, limiting the tumor from growing beyond a size of more than 1 mm in diameter and subsequently forcing it to enter a dormant state [83]. To sustain sufficient growth support, cancer cells promote vascularization by inducing angiogenesis, another hallmark for tumor development [83]. Normally, angiogenesis occurs during wound healing [83]. A fine-tuned balance of angiogenic activators and inhibitors controls the production of new blood vessels [83]. However, tumor cells can induce the angiogenic switch by tipping this balance to the pro-angiogenic state [83]. Compared to normal blood vessels, tumor vasculature is organized chaotically, resulting in abnormal blood flow and leaky vessels, which increase the likelihood of distant metastases [83]. Two lncRNA-encoded microproteins display opposing roles during angiogenic development in TNBC, including ASRPS and XBP1SBM [16,84]. ASRPS inhibits angiogenesis by regulating the VEGF pathway through its interaction with STAT3, whereas XBP1SBM can upregulate VEGF expression level, promoting angiogenesis in TNBC (Figure 3) [16,84]. 

### 2.3. Inflammation and Immune Responses

Tumor cells express specific antigens that immune cells recognize as non-self and targeted for destruction [85]. Antigen-presenting cells (APC) such as dendritic cells (DCs) recognize and display tumor-specific antigens, which elicit T-cell responses to destroy tumor cells [85]. However, tumor cells exploit various mechanisms to evade immune surveillance, such as downregulating antigens or inducing an immunosuppressive microenvironment [85]. A common strategy is to induce immune tolerance to cancer by expressing the ligands for the receptor of the Ig superfamily, PD-1 [85]. 

Advancements in proteomics technologies allow the identification of peptides or proteins derived from ncORFs. Several ncORF-encoded microproteins contribute to immune responses (Figure 3). For example, the identification of non-annotated ORFs led to the discovery of PC3-secreted microprotein (PSMP), a chemoattractant protein highly expressed in some prostate cancers. PSMP regulates inflammation during tumor development through binding to the CCR2B receptor and activates downstream ERK/MAPK signaling (Figure 3) [86]. Moreover, protein-coding ORFs within lncRNA Aw112010 express proteins essential for mucosal immunity [87]. Another microprotein encoded by a non-protein coding transcript is miPEP155, which suppresses DC-driven auto inflammation and T-cell priming by regulating antigen presentation through disrupting HSP70–HSP90 interaction (Figure 3) [88]. Deep mining of non-canonical proteins also results in the discovery of tumor-specific peptides, highlighting their potential for establishing cancer immunotherapies [89,90]. 

### 2.4. DNA Damage Response and Genetic Instability

Genomic integrity relies on DNA monitoring and repair mechanisms to ensure a pristine state of the whole genome [56]. Genomic DNA is sensitive to exogenous and endogenous damage, resulting in genetic instability [91]. Diverse DNA damage response mechanisms have evolved to sustain the genome integrity, including canonical homologous recombination repair (HRR), non-homologous end-joining (NHEJ) pathways, and the post-replication repair (PRR) pathway [91]. Yet, tumor cells accumulate substantial genomic alternations that allow them to acquire survival advantages during carcinogenesis [56]. Defects of DNA repair mechanisms explain the high mutational rate found in cancer cells [56]. Genomic instability enabled by malfunctions of DNA repair machinery then promotes clonal diversity and selection [56]. Known microproteins regulating genome stability include MRI-2, which interacts with DNA end-binding protein Ku in the nucleus to stimulate the NHEJ DNA repair pathway (Figure 3) [92]. PACMP and DDUP are reported lncRNA-encoded microproteins that also trigger DNA damage responses through different mechanisms [91,93]. PACMP modulates DDR by preventing degradation of CTBP-interacting protein and enhancing PARP1-dependent poly(ADP-ribosyl)ation (Figure 3) [93]. DNA damage induces upregulation of DDUP, which regulates PCNA ubiquitination and RAD18 retention at DNA damage sites, inducing HRR and PRR mechanisms [91]. The ubiquitin-like microprotein TINCR is reported to be a protective factor preventing UV exposure as accelerated skin lesions were observed in UV-treated mice with TINCR mutant [69]. 

### 2.5. Metabolism

Reprogramming of cellular metabolism is another core hallmark of cancer development, supporting the acquisition and maintenance of malignant phenotypes [94]. Anabolic growth in nutrient-depleted conditions is made possible by altering metabolic activity in cancer cells for survival [94]. The Warburg effect or aerobic glycolysis is a classic example of metabolic reprogramming found in cancer [94]. In the 1920s, Warburg discovered that cancer cells constitutively uptake glucose and convert it into lactate, regardless of the presence of oxygen [94]. Elevated glycolytic intermediates are utilized as macromolecule precursors to fulfill the demands of proliferating cells [94]. Additionally, intermediates from the tricarboxylic acid cycle can be supplied into subsidiary pathways for macromolecule synthesis [94]. Thus, the ability of cancer cells to reprogram metabolic pathways supports tumorigenic proliferation and progression. The selective expression of glycolytic enzyme pyruvate kinase M (PKM) isoforms is regulated by splicing factors, such as hnRNP A1 [62]. Re-expression of PKM2 in human cancers is thought to promote aerobic glycolysis, enabling proliferative advantages [62]. A small peptide encoded by the lncRNA *HOXB-AS3* suppresses glucose reprogramming by antagonizing hnRNP A1-mediated PKM splicing in CRC (Figure 3) [62]. Lactate utilization is restricted by MP31, a microprotein encoded from 5′ UTR of PTEN in GBM [95]. Loss of MP31 increased global lactate metabolism, enhancing tumorigenicity [95]. 

Abnormal lipid oxidation is prevalent during tumor development [96,97]. In addition to glucose, tumor cells adapt to the nutrient-deprived microenvironment by increasing fatty acid metabolism [96]. A key enzyme of fatty acid oxidation (FAO), carnitine palmitoyl transferase 1A (CPT1A), is upregulated by an exosomal lncAKR1C2-encoded microprotein, pep-AKR1C2, inducing a metabolic switch towards FAO in gastric cancer (Figure 3) [96,98]. Lipid metabolism and homeostasis can be influenced by microproteins, including CASIMO1 and ACLY-BP, through distinct mechanisms, enhancing proliferation in BC and ccRCC, respectively (Figure 3) [59,65]. 

## 3. ncORFs in Cancer Diagnosis and Prognosis

**Figure 4:**
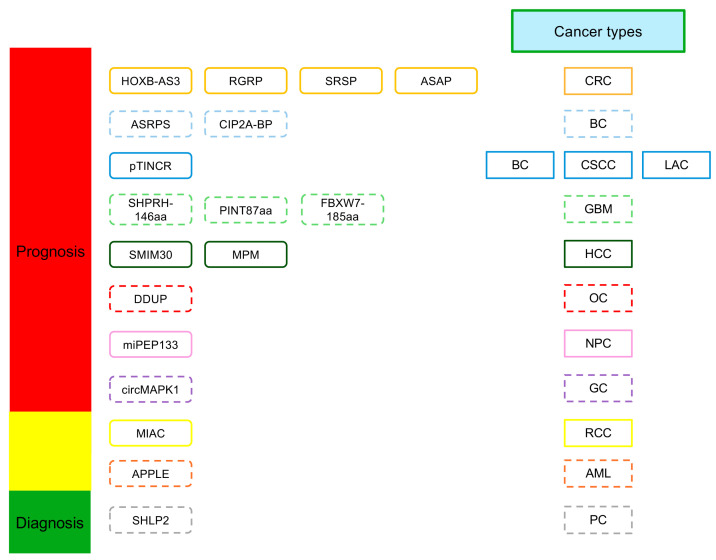
ncORFs as prognosis and diagnosis biomarkers. The prognostic and diagnostic roles of microproteins encoded by ncORFs in different human cancers is shown with dashed lines. AML, acute myeloid leukemia; BC, breast cancer; CRC, colorectal carcinoma; CSCC, cutaneious squamous cell carcinoma; GBM, glioblastoma; GC, gastric cancer; HCC, hepatocellular carcinoma; LAC, lung adenocarcinoma; NPC, nasopharyngeal carcinoma; OC, ovarian cancer; PC, prostate cancer; RCC, renal cell carcinoma.

Selective expression of certain ncORFs has been observed in specific cancers and is associated with worse disease outcomes, enabling early detection and progression prediction of certain diseases [99,100,101,102,103]. Here, we discuss known non-canonical proteins and their involvements as diagnostic and prognostic factors (Figure 4). 

### 3.1. Colorectal Cancer

HOXB-AS3 is a lncRNA that plays an oncogenic role in human cancers and displays an elevated RNA expression in the cancer cells [104,105,106,107]. Interestingly, Huang et al. (2017) discovered that HOXB-AS3 encodes a small peptide, and they found that the expression of HOXB-AS3 RNA and the peptide was downregulated in colorectal cancer (CRC) tissues compared to paired adjacent normal tissues [62]. Furthermore, the tumor suppressive effects of the HOXB-AS3 peptide were revealed, and a low level of the HOXB-AS3 peptide was associated with worse prognosis of CRC patients [62]. Specifically, Kaplan–Meier survival analyses indicated that CRC patients with high HOXB-AS3 peptide expression had a 1.6-fold increase in mean overall survival time compared to those with low expression, suggesting that high expression of the HOXB-AS3 peptide correlates with reduced CRC-related mortalities [62]. The expression of SRSP, another lncORF-encoded peptide, was significantly elevated in CRC tissues compared to normal colorectal tissues [108]. Further investigation revealed that SRSP expression was positively associated with clinicopathological features of CRC patients, such as histological grade, presence of lymph node metastasis, clinical stage, and risk of death [108]. CRC patients with upregulated SRSP expression had 3.3-fold reduction in median survival time compared to those with low expression [108]. Other examples of potentially prognostic or diagnostic ncORF-encoded peptides in CRC include RBRP and ASAP, derived from lncRNAs, and PPP1R12A-C, derived from circRNA [15,64,109].

### 3.2. Breast Cancer

ASRPS, a lncORF-encoded peptide, is significantly downregulated in triple-negative breast cancer (TNBC) compared to other breast cancer subtypes, and low ASRPS expression is correlated with worse prognosis and overall survival in TNBC patients [16]. The low expression of another lncORF-encoded peptide, CIP2A-BP, showed a similar association with poorer prognosis in TNBC [18]. Boix and colleagues (2022) demonstrated that the lncORF-encoded peptide pTINCR was upregulated in epithelial tissue upon cellular stress and promoted epithelial differentiation in several cancers of epithelial origin, including luminal breast cancer [70], cutaneous squamous cell carcinoma, and lung adenocarcinoma. Although pTINCR peptide expression was not analyzed, correlation studies using public data revealed that high TINCR lncRNA expression was associated with more favorable prognoses in various epithelial cancers including breast cancer, suggesting the pTINCR peptide may be prognostically significant [70].

### 3.3. Glioblastoma

SHPRH-146aa is a circORF-encoded peptide that has the potential to serve as a prognostic or diagnostic biomarker for glioblastoma (GBM) [110]. The expression of SHPRH-146aa was downregulated in GBM tissue samples, and longer patient survival times were correlated with higher SHPRH-146aa expression [110]. PINT87aa, another circORF-encoded peptide, displayed a similar association with clinical prognosis of GBM [68]. When comparing GBM of varying WHO grades and normal brain tissues, PINT87aa expression was significantly reduced in GBM tissues and negatively correlated with tumor grade, with WHO grade IV glioblastomas displaying the lowest PINT87aa expression [68]. Another circORF-encoded peptide, FBXW7-185aa, also exhibited reduced expression and positive correlations with survival times of GBM patients [66]. 

### 3.4. Hepatocellular Carcinoma (HCC)

In HCC, the lncORF-encoded peptide SMIM30 has been associated with poor patient survival rates [60]. MPM, also a lncORF-encoded peptide, was significantly downregulated in HCC tissues, with low MPM expression associated with increased HCC metastasis, recurrence, and mortality [111]. 

### 3.5. Ovarian Cancer (OC)

DDUP is a peptide encoded within the lncRNA CTBP1-DT and is involved in DNA damage response (DDR) signaling [112]. Ren and colleagues (2023) demonstrated that high CTBP1-DT expression conferred markedly shorter overall and progression-free survival in ovarian, lung, and gastric cancer patients [112]. Notably, it has been shown that the DDUP peptide, not the CTBP1-DT lncRNA, promoted chemoresistance in patient-derived ovarian cancer cells [112]. 

### 3.6. Prostate Cancer

Although these results emphasize that ncORFs and their encoded peptides have potential clinical diagnostic and prognostic values in various human cancers, much of their detection occurs in the context of biopsied tissue samples. If these peptides can be detected non-invasively, such as in body fluids like blood, serum, or urine, it will immensely benefit their adoption as cancer biomarkers in clinical settings [99]. An example of such an ncORF is SHLP2, a mitochondrial-derived peptide that has demonstrated potential diagnostic significance in prostate cancer (PC) [113]. Xiao and colleagues (2017) analyzed serum SHLP2 concentrations in black and white PC patients and found SHLP2 expression to be significantly reduced in white confirmed PC cases compared to white healthy controls, while no significant difference in SHLP2 expression could be observed in black cases vs. controls. They have shown that downregulation of SHLP2 peptide expression was associated with greater PC risk in white men, and high serum SHLP2 concentration accurately ruled out PC in both racial groups, demonstrating potential as a novel diagnostic biomarker for PC [113]. Moreover, no association was found between SHLP2 expression and PC clinical grade, suggesting that SHLP2 did not confer any prognostic value [113]. However, the diagnostic value of SHLP2 requires further validation since a relatively small dataset was analyzed in the study. 

### 3.7. Other Types of Cancer

miPEP133, a pri-miORF-encoded peptide, was demonstrated by Kan and colleagues (2020) to be a favorable prognostic biomarker for nasopharyngeal carcinoma (NPC) [75]. Low miPEP133 mRNA levels were associated with advanced metastatic progression in NPC and significantly worse overall survival in NPC patients [75]. 

Sun and colleagues demonstrated that lncRNA ASH1L-AS1 encoded a peptide called APPLE, and both the lncRNA and the peptide were significantly upregulated in preliminary acute myeloid leukemia (AML) patients when compared to healthy controls or AML patients in complete remission [17]. AML patients with high ASH1L-AS1 expression were more likely to achieve a 5-year leukemia-free survival than those with low expression [17]. Interestingly, ASH1L-AS1 expression could also be used to differentiate patients in complete remission status and those with preliminary diagnosis, further supporting the status of ASH1L-AS1 and APPLE as predictors of poor AML prognosis [17]. 

circMAPK1, a circORF-encoded peptide, exhibited downregulated expression in gastric cancer (GC) tissue compared to paired adjacent normal tissues [32]. High circMAPK1 expression was also linked with favorable clinicopathologic parameters (e.g., tumor size, lymphatic invasion, clinical stage) as well as longer overall survival [32]. 

MIAC, a lncORF-encoded peptide, was demonstrated to have higher expression in renal cell carcinoma (RCC) tissue compared with normal tissues in TCGA database screening as well as clinical samples [114]. RCC patients with high MIAC expression demonstrated significantly higher survival rates than those with low expression [114]. MIAC expression was also significantly higher in patients with early stage renal cancer than those in advanced stages [114]. This result emphasizes that MIAC lncORF has potential clinical diagnostic and prognostic value in RCC. 

To identify novel prognostic and diagnostic biomarkers for cancer, proteomic approaches can be applied. For example, in a recent study, global micro-/alt-protein quantitation in two human leukemia cell lines, K562 and MOLT4, identified 12 unannotated proteins that were differentially expressed in these cell lines [115]. Further quantification of ncORF protein in clinical samples will identify novel ncORF biomarkers for the diagnosis and prognosis of human cancers.

## 4. ncORF in Cancer Therapy

### 4.1. ncORF in Anti-Cancer Drug Response

**Figure 5:**
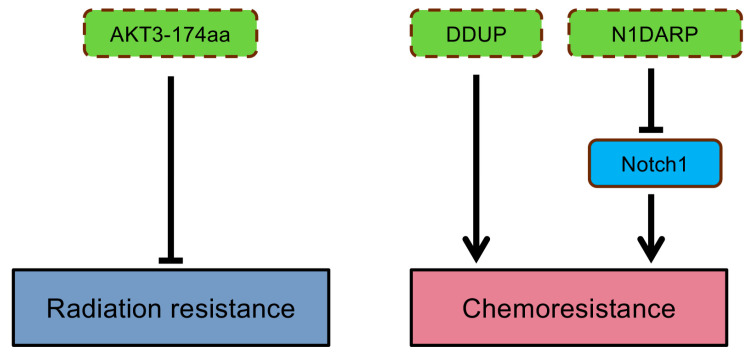
The contribution of ncORFs-encoded microproteins in anti-cancer drug responses. Labels are as described in legend of Figure 2.

Drug resistance occurs when cancer cells tolerate a treatment, greatly limiting the efficiency of anti-tumor therapies in many patients [116,117]. Genomic instability is one factor contributing to drug resistance, which can be intrinsic or acquired [117]. Innate resistance exists in patients even before administrating anti-cancer drugs due to pre-existing genetic perturbations, tumor heterogeneity, or activation of defense mechanisms [117]. For example, resistance to cisplatin treatment was found in gastric cancer patients with HER2 overexpression, which upregulated the expression of EMT-related transcriptional factor Snail [118]. Moreover, studies show that Snail and Slug not only mediate EMT but also promote stem-like characteristics, conferring resistance to radiotherapy and chemotherapy [118,119]. Alternatively, relapse to treatment can occur when new mutations are acquired in tumor cells, resulting in a gradual decrease in therapy efficacy [117]. For instance, a point mutation from threonine-to-isoleucine in the BCR-ABL kinase domain can cause resistance or relapse of tyrosine kinase inhibitor treatment in patients with chronic myelogenous leukemia [120]. Several microproteins have been shown to affect drug efficiency in cancers (Figure 5). Radiation sensitivity was enhanced by overexpressing AKT3-174aa in GBM cells, while loss of AKT3-174aa expression regained resistance to radiation [67]. Chemoresistance can be acquired by evading DNA-damage-induced genetic instability [117]. DDUP is a lncRNA-encoded microprotein and functions as a regulator of DDR, promoting resistance to DNA-damaging chemotherapies [91]. Inhibition of DDUP by the ATR inhibitor Berzosertib re-sensitized ovarian cancer cells to cisplatin treatment, indicating its vital role in affecting drug response [91]. The tumor-suppressive role of a microprotein, N1DARP, was recognized in pancreatic cancer since it could suppress Notch1 signaling pathways, which contribute to chemoresistance [121].

### 4.2. ncORFs as Potential Drug Targets for Cancer Therapy

Given the fact that ncORFs or microproteins play important roles in various human diseases including cancer, their roles as therapeutic targets have been proposed or demonstrated recently [122]. Targeted therapy is a high-precision treatment designed to attack certain molecules that play oncogenic roles in tumors [99]. Its goal is to minimize non-specific cytotoxicity while destroying the growth and survival of cancer cells [99]. Molecular targeted therapeutic agents act on growth factors, cell surface antigens, and signaling pathways that initiate and maintain cancer hallmarks to block cancer progression [116]. Many anti-cancer therapies target different anti-apoptotic components, such as Bcl-2 family proteins, to induce apoptosis in tumor cells [116]. Molecular targeted therapies offer significant advantages in treating different types of cancers, including breast, gastric, colon, and lung cancers [116]. Compared to conventional chemotherapy, inhibitors of EGFR-tyrosine kinase improve clinical outcomes in NSCLC patients with EGFR mutations [116]. Despite the observed advantages, limitations exist and restrict the efficiency of targeted therapy in cancer patients [116]. Since targeted treatment requires specific biomarkers to be effective, patients who do not express such targets will respond poorly [116]. Several treatment options have been proposed for cancer patients [98]. Cancer cells are enriched with ncORF MHC-I-associated peptides (MAPs), suggesting their potential as a source of tumor antigens for immunotherapeutic applications. Additionally, pharmacological targeting of cancer-specific ncRNAs to silence pro-oncogenic microprotein translation may lead to downstream inhibition of previously undruggable targets. Microproteins can also provide valuable insights for drug design by identifying protein–protein interaction hotspots or inhibitor binding sites. Exploring microprotein conjugation to other drugs may offer potential applications in molecular glues or heterobifunctional compounds. Microproteins are also promising candidates as non-antibody binding scaffolds. Furthermore, the differential expression of ncORFs and microproteins in cancer enhances their potential as biomarkers, particularly in cases involving differentially expressed secreted microproteins.

## 5. Conclusions and Future Perspective

The recognition of ncORFs has attracted increasing attention and uncovers new aspects of regulating biological processes. The protein-coding capacity of the human genome is greatly underestimated and remains to be fully explored. Improved approaches prompt the identification of many ncORF-encoded peptides involved in cancer pathogenesis. Even though an increasing number of proteins expressed from ncORFs are unveiled, only a small set of these peptides are functionally characterized. Regulatory functions of many microproteins translated from ncRNAs are reported to influence tumor progression. Many ncRNA-derived microproteins can act as tumor suppressors or inducers. Thus, understanding the role of these ncRNA-encoded proteins offers critical prospects for cancer therapies. 

In this review, we describe different types of ncRNAs reported to have coding potential, mention present techniques used for capturing ncORFs, and summarize functional proteins encoded by ncORFs implicated in cancer research. The discovery of ncORF-encoded microproteins expands our understanding of tumor development as many of them display prognostic and/or carcinogenic roles in human cancers. Despite more and more studies focusing on ncORFs, only a small portion of ncRNA-encoded microproteins have been identified and validated, and their mechanisms of action in cancer remain mostly unclear. Therefore, identifying and characterizing noncanonical proteins may lead to the establishment of novel biomarkers and drug targets for cancer diagnosis and therapeutics. 

## Data Availability

Not applicable.
